# Bio-tribological behavior of articular cartilage based on biological morphology

**DOI:** 10.1007/s10856-021-06566-y

**Published:** 2021-10-22

**Authors:** Xinyue Zhang, Yi Hu, Kai Chen, Dekun Zhang

**Affiliations:** 1 School of Mechatronic Engineering, China University of Mining and Technology, Xuzhou, Jiangsu 221116 People’s Republic of China; 2 School of Materials and Physics, China University of Mining and Technology, Xuzhou, Jiangsu 221116 People’s Republic of China

## Abstract

Artificial hemiarthroplasty is one of the effective methods for the treatment of hip joint diseases, but the wear failure of the interface between the hemi hip joint material and articular cartilage restricts the life of the prosthesis. Therefore, it is important to explore the damage mechanism between the interfaces to prolong the life of the prosthesis and improve the life quality of the prosthesis replacement. In this paper, the creep and bio-tribological properties of cartilage against PEEK, CoCrMo alloy, and ceramic were studied, and the tribological differences between “hard–soft” and “soft–soft” contact were analyzed based on biomorphology. The results showed that with the increase of time in vitro, the thickness of the cartilage membrane decreased, the surface damage was aggravated, and the anti-creep ability of cartilage was weakened. Second, the creep resistance of the soft–soft contact pair was better than that of the hard–soft contact pair. Also, the greater the load and the longer the wear time, the more serious the cartilage damage. Among the three friction pairs, the cartilage in PEEK/articular cartilage was the least damaged, followed by CoCrMo alloy/articular cartilage, and the most damage was found in ceramic/articular, indicating that the soft–soft friction pair inflicted the least damage to the cartilage.

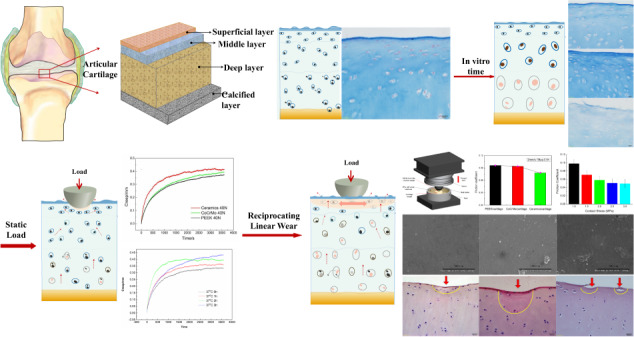

## Introduction

At present, osteoarthritis caused by articular cartilage wear has become the most common disabling disease in the world, with about 400 million people suffering from it. Artificial joint replacement is an effective method to reconstruct joint function and treat arthritis, which can be divided into hemiarthroplasty and total hip arthroplasty. For young patients with osteonecrosis of the femoral head, doctors mainly advocate hemiarthroplasty [1], but with the increase of service life, the acetabular cartilage will undergo progressive degeneration due to cartilage wear and patients often need revision surgery. To reduce cartilage wear and increase the service life of artificial joints, it is necessary to study the tribological properties of natural articular cartilage and hemi hip joint materials [2].

Natural joints are “soft–soft” contact pairs due to the natural cartilage, a highly hydrated biological tissue covering the joint surface, that can effectively reduce shock impact, absorb shock, reduce the impact of joint movement, and lower impact abrasion [3]. The common artificial hemi hip replacement materials are CoCrMo alloys and zirconia ceramics, forming “hard–soft” contact pairs with cartilage, resulting in severe cartilage wear, osteolysis, aseptic loosening, and prosthesis failure [4–6], leading to a gap with natural joints. Qian and Shirong [7] used natural articular cartilage/ceramics to carry out a tribological test on reciprocating motion tests to explore the wear of hemi hip replacements. The results showed that the form was mainly abrasive wear. Vanlommel et al. [8] compared the damage of porcine patellar cartilage by OxZr and CoCr, and the results showed that both implants could cause cartilage surface degradation. Wimmer et al. [6] evaluated the effect of an alumina–zirconia composite (AZC) as a joint interface against living cartilage graft, and compared with clinical-related CoCrMo; AZC induced less cell and tissue damage than CoCrMo. Stojanovic et al. [9] studied the friction corrosion effect of the interface of CoCrMo alloys and cartilage during the sliding process, indicating that the release of metal ions and particles will form compounds with potential cytotoxicity to cartilage tissue.

Polyetheretherketone (PEEK), a new prosthesis material, has become the most promising artificial bone matrix composite because of its excellent wear resistance, biocompatibility, and chemical stability [10, 11]. Also, PEEK with lower hardness and elastic modulus is closer to human bone, which can achieve soft–soft contact with joints to some extent. Lee et al. [12] indicated that implants made of PEEK are more in line with fatigue strength requirements, and their elastic modulus is close to that of cortical bone, which can effectively reduce the effect of stress shielding. The fretting corrosion behaviors of Ti6Al4V/PEEK and Ti6Al4V/CoCrMo were studied by Xu et al. [13], indicating that Ti6Al4V/PEEK has better corrosion resistance. Liu et al. [14] carried out a torsion friction test of PEEK and CoCrMo alloys, and the results showed that PEEK showed a low friction coefficient under different conditions.

Most studies focus on the surface wear morphology and wear amount, but it is relatively difficult to characterize the wear evaluation of cartilage. Also, there is little study of the evolution of cartilage after wear and the relationship among cartilage morphology, material friction, and wear based on biological morphology. Herein, a HE staining method was used to explore the tribological characteristics of articular cartilage. The wear mechanism of cartilage and the relationship among cartilage morphology, friction, and wear under different joint friction pairs, loads, and time were analyzed. The tribological differences between the hard–soft and soft–soft contact pairs were compared, which provided a theoretical basis for the selection of hemi hip joint materials.

## Experiment

### Materials

Natural articular cartilage was obtained from an 18-month-old adult femur, obtained within 4 h after slaughter, stored in physiological saline within 36 h, and stored frozen in a −20 °C cryostat for testing. For cartilage samples with different times in vitro, frozen spare articular cartilage was thawed at room temperature and stored in 37 °C incubators for 1, 2, and 3 h. The flat area of the cartilage center was selected to be processed into a disc ∅ 25 × 8 mm by using a ∅ 25 mm hollow drill bit, and then the lower surface cut and polished to ensure a level of the bottom surface of the sample. PEEK, CoCrMo alloy, and zirconia ceramic were selected as accessory materials of cartilage in a 10 mm diameter ball. PEEK was purchased from Jiangsu Aokangni Medical Technology Development Co., Ltd.; CoCrMo alloy and ceramics were purchased from Shanghai Shibo Metal Products Co., Ltd. The material properties are shown in Table 1.

**Table 1:** Physical and mechanical properties [2, 20]

Materials	Tensile strength (MPa)	Density (kg/m^3^)	Elastic modulus (GPa)	Poisson ratio	Vickers hardness
PEEK	97	1300	3.6	0.35	30–40
CoCrMo	970	8500	240	0.3	321
Cartilage	113	1800	0.0105	0.1	–
Ceramics	960	4370	220	0.35	1200

## Methods

### Creep test

**Fig. 1:**
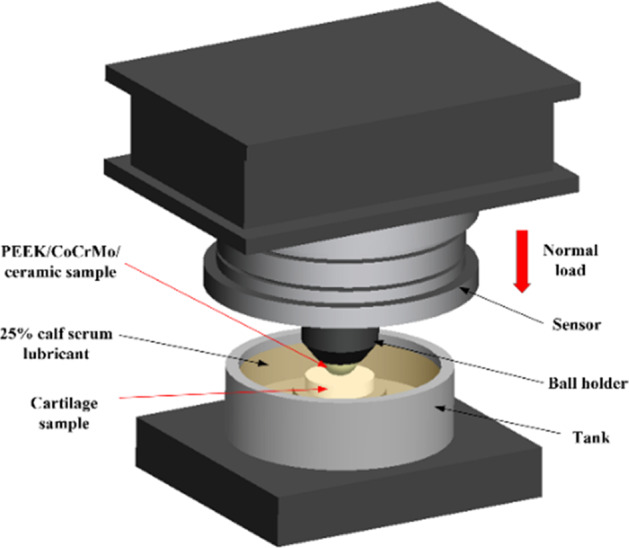
The creep experimental device schematic

The effects of different times in vitro and different contact pairs on the creep behavior of articular cartilage were investigated using the friction and wear tester (Purchased from Rtec Instrument Technology Co., Ltd). The experimental parameters are shown in Table 2, and the schematic diagram of the experimental device is shown in Fig. 1.

**Table 2:** Creep test parameters

No.	In vitro time (h)	Contact pairs	Lubricating medium	Normal load (N)	Test time (h)
1	0/1/2/3	PEEK-cartilage	25% calf serum	40	1
2	0	PEEK/CoCrMo/ceramics-cartilage

### Tribological test

**Fig. 2:**
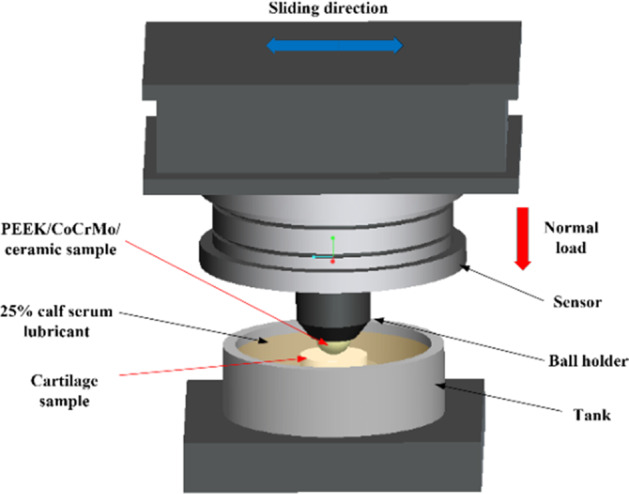
Sliding friction experimental device schematic

The effects of friction pairs, normal loads, and friction time on the tribological behavior of joint material were investigated using the friction and wear tester (Rtec Instrument Technology Co., Ltd. (Nanjing)). The experimental parameters are shown in Table 3, and a schematic of the experimental device is shown in Fig. 2.

**Table 3:** Experimental parameters of bio-friction

No.	Contact pairs	Normal stress	Wear time (h)	Lubricating medium	Sliding velocity (mm/s)
1	PEEK/CoCrMo/ceramics-cartilage	1	0.5	25% calf serum	2
2	PEEK/cartilage	1/1.5/2/2.5/3	0.5		
3	PEEK/cartilage	1	1/2/6/12

### Biomorphology test

HE staining was used to characterize the biological morphology of articular cartilage to explore the effects of time in vitro, creep, and friction behavior on the morphology of articular cartilage. First, the specimen with a size of 10 * 10 * 4 mm was taken from the prepared articular cartilage. Second, the sample was fixed with Bouin solution, dehydrated with ethanol, transparent with xylene, embedded in paraffin, and cut with a slicer to 8 μm. Third, the slices were immersed in xylene for 10 min, then the slices were successively treated with ethanol (100, 95, 85, and 70%), and then immersed in distilled water for 5 min to remove paraffin. Last, HE staining was performed and observed under the microscope. For HE staining, the chromatin in the nucleus and nucleic acids in the cytoplasm appears purple-blue and the cytoplasm and the extracellular matrix appear red [15].

## Results and discussion

### Biomorphology

**Fig. 3:**
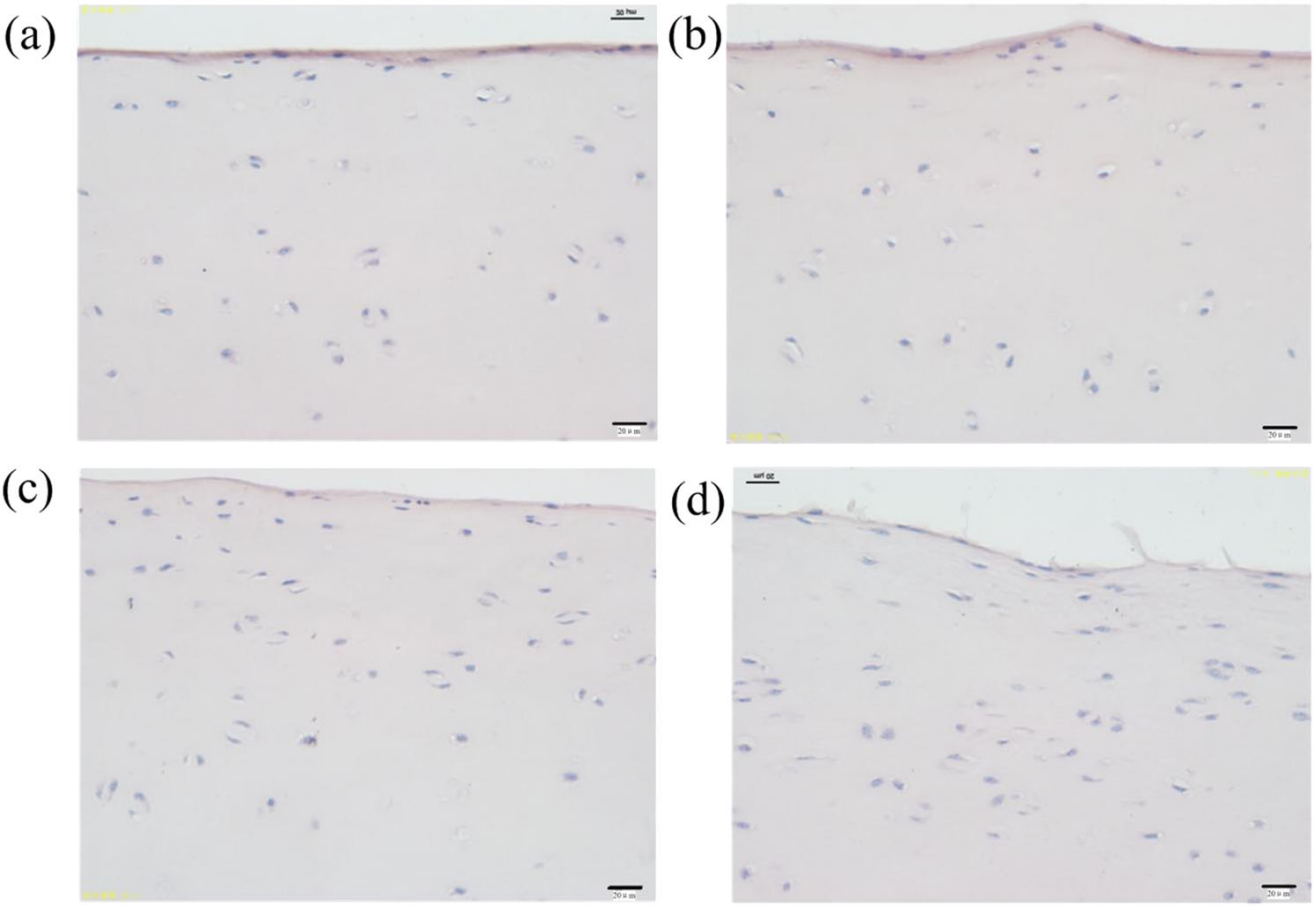
HE staining morphology of articular cartilage at different times in vitro. **a** Fresh bovine cartilage; **b** 37 °C incubator for 1 h; **c** 37 °C incubator for 2 h; **d** 37 °C incubator for 3 h

Figure 3 shows the HE staining morphology of articular cartilage at different times in vitro. It can be seen that as the time in vitro increases, the thickness of the cartilage membrane gradually decreased and cartilage surface damage increased. For fresh articular cartilage (Fig. 3a), the cartilage membrane is even and deeply stained with a thickness of 6.20 ± 0.60 μm. There is a clear boundary between the cartilage membrane and the deep part of the cartilage. The nucleus is round or fusiform (osteogenitor cell), and the cells closer to the perichondrium are smaller in size and oblate in shape with a long axis parallel to the cartilage surface; the farther away from the cell membrane, the larger the nucleus. In the 37 °C incubator for 1 h (Fig. 3b), the surface of cartilage was uneven with shallow staining and thin perichondrium, and cartilage nuclei became fuzzy. After 2 h (Fig. 3c), the thickness and staining of cartilage membrane decreased significantly, and the number of chondrocytes with fuzzy nuclei increased. After 3 h (Fig. 3d), the perichondrium was severely exfoliated with the thinnest thickness (1.52 ± 0.42 μm) and shallowest staining. The cartilage matrix was dissolved, the cartilage nucleus was fuzzy with common nuclear vacuoles, and many chondrocytes were desalted or disappeared.

The thickness of the cartilage film decreased with increased in vitro time, as determined by the composition of articular cartilage. The main components of articular cartilage include water, chondrocytes, collagen fibers, proteoglycans, and extracellular matrix, of which more than 75% is water. With the increase in time, the water in articular cartilage is continually lost, so the articular cartilage membrane thins.

### Creep

**Fig. 4:**
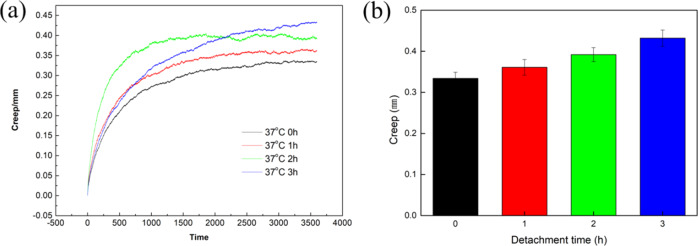
Creep of articular cartilage at different times in vitro. (**a**) time-dependent curve of creep, (**b**) creep at last stage

Figure 4 shows the creep of articular cartilage at different times. It can be seen that the peristalsis of fresh cartilage was 0.334, 0.361, 0.392, and 0.432 mm, respectively, after 0, 1, 2, and 3 h of placement, which showed that the anti-creep ability of cartilage decreased with increased time in vitro. With the increase of in vitro time, the water loss makes the liquid-phase-carrying capacity drop sharply, at this time, the consumption of collagen fiber and proteoglycan increases, and the strain increases. This requires that the artificial joint replacement should be controlled as long as possible to reduce the amount of bleeding and maintain cartilage activity.

**Fig. 5:**
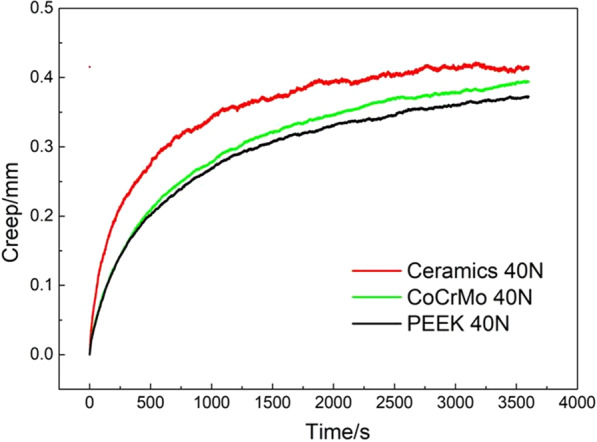
Creep curves of different contact pairs

Figure 5 shows the creep of natural cartilage in different contact pairs. The normal strains of PEEK/articular, CoCrMo/articular, and ceramic/articular cartilage were 0.372, 0.394, and 0.414 mm. Also, the strain rate of the three pairs was similar, which showed that the strain rate increased rapidly in the initial stage and then became flat, but in the initial stage (0–500 s), the strain rate of cartilage in ceramic/articular cartilage was the fastest, followed by CoCrMo alloy/articular cartilage, and peek/articular cartilage. This was because the elastic modulus and hardness of ceramic and CoCrMo alloy were much higher than PEEK, and PEEK is more prone to viscoelastic deformation. Therefore, compared with hard–soft matching, cartilage deformation in soft–soft matching was smaller.

### Tribological behavior

**Fig. 6:**
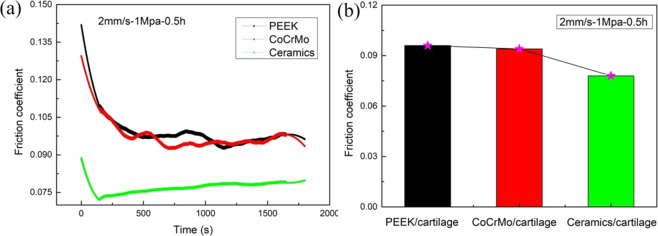
The friction coefficient of different friction pairs. **a** Time-varying friction coefficient; **b** stable friction coefficient

Figure 6 shows the friction coefficient of different friction pairs. From the time-varying friction coefficient (Fig. 6a), it can be seen that in the initial stage, the contact of the friction pair is unstable and the friction coefficient is large due to the micro concavity, and then with the friction, the contact of pairs tends to be stable. Also, the natural cartilage has two phase-loading characteristics. In the initial stage, the hydraulic loading mechanism of cartilage lags because of the influence of the viscoelasticity of cartilage, which makes the initial friction coefficient larger. But with the increase of friction time, the hydraulic loading mechanism of cartilage gradually responds, and the soft bone surface is evenly loaded, which makes the friction coefficient gradually stable. The friction coefficients of the PEEK/articular cartilage and CoCrMo alloy/articular cartilage friction pairs change similarly, with the friction coefficients decreasing sharply and then stabilizing. The friction coefficients of ceramic/articular cartilage decrease sharply at first, then increase slowly and stabilize.

It can be seen from Fig. 6b that the friction coefficients of ceramic/articular cartilage decrease significantly compared with the others. This is because the friction coefficient is closely related to the tissue characteristics of the material. The hardness, strength, modulus of elasticity, and other parameters affect the friction coefficient of the material. The hardness of the ceramic is far greater than that of the other two materials. During the wear process, the surface damage of the ceramic is slight, and the micro-convex body on the cartilage surface is gradually flattened under the complex friction such that the surface contact area increases, and the friction coefficient is minimal. It can be seen from the creep characteristics of the three pairs in Fig. 5 that under the same load, the normal deformation of ceramic/articular cartilage is the largest. In other words, the actual contact area of ceramic/articular cartilage is the largest, that of CoCrMo alloy/articular cartilage is second, and that of PEEK/articular cartilage is the smallest.

**Fig. 7:**
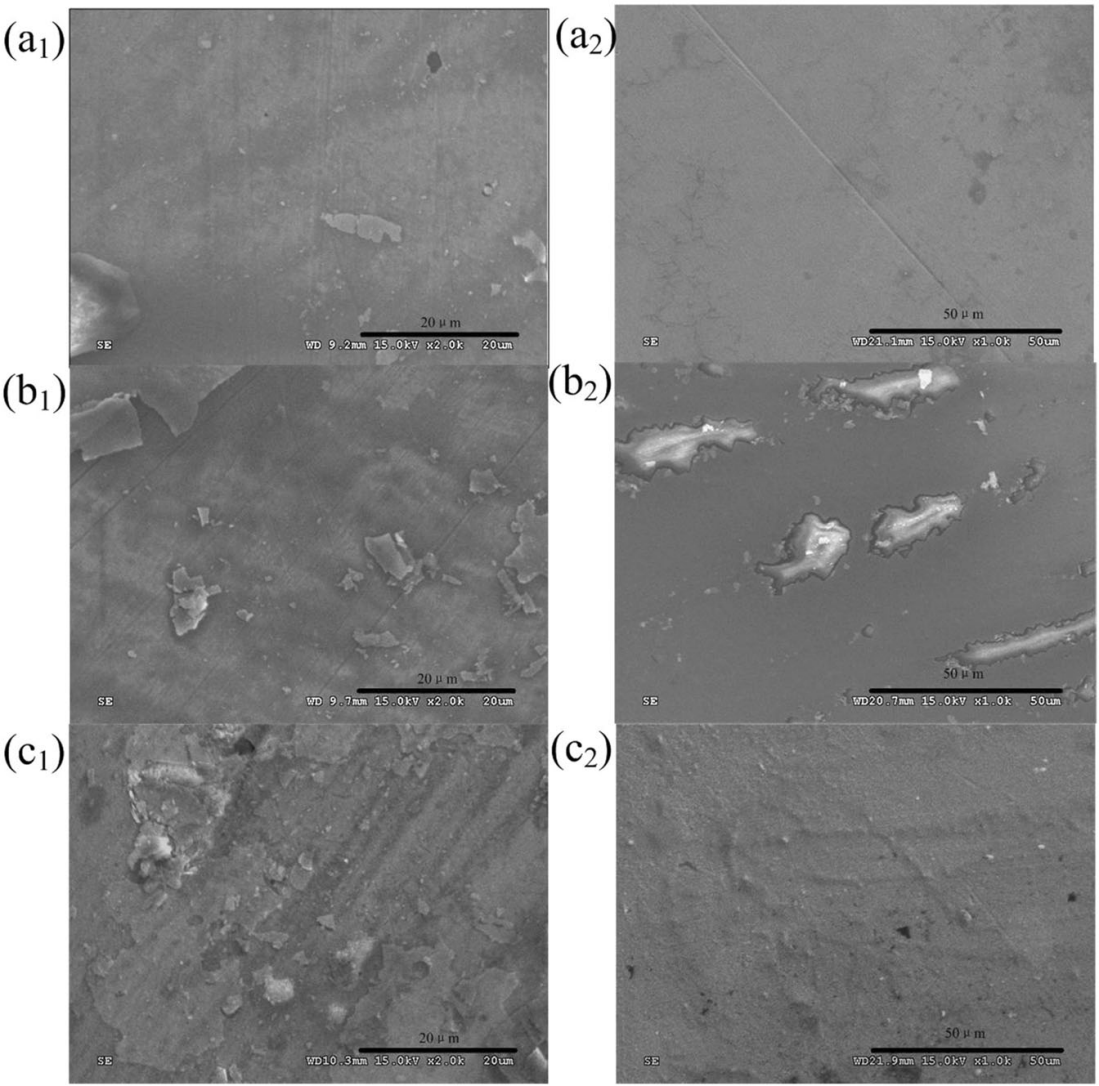
SEM of different pins. **a1** PEEK before cleaning; **a2** PEEK after cleaning; **b1** CoCrMo before cleaning; **b2** CoCrMo after cleaning; **c1** ceramic before cleaning; **c2** ceramic after cleaning

Figure 7 shows the surface morphology of CoCrMo, PEEK, and ceramic pins before and after cleaning. It can be seen from the SEM of the samples before cleaning (a1, b1, c1), there are adherents on the surfaces of the three pins. The PEEK surface is relatively uniform with small and single adherents, the CoCrMo surface is attached with a thick layer and irregular flakes, and the ceramic surface accumulates some agglomerates. From the surface morphology (a2, b2, c2) after cleaning, it can be observed that the attachments on the three materials’ surfaces have been reduced. The attachment should be a mixture of worn-out proteins on the cartilage surface and macromolecular proteins in calf serum. The surface of the PEEK is slightly damaged, accompanied by a small number of deep scratches and micro-convex bodies. There are discontinuous wear marks on the surface of CoCrMo, and there are adhesion accumulation and tear pits between the wear marks. The ceramic surface is the most uneven, with long ridge accumulation perpendicular to the scratch direction, and the scratches are relatively dense. The results show that the selection of friction pairs plays an important role in reducing wear.

**Fig. 8:**
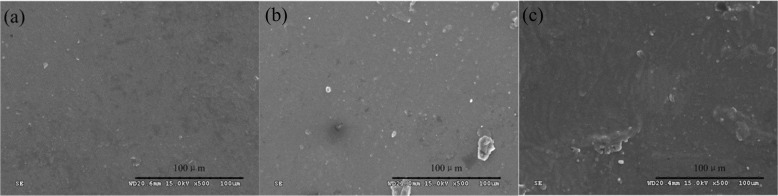
The SEM of cartilage in different pairs. **a** PEEK/cartilage; **b** CoCrMo/cartilage; **c** ceramic/cartilage

Figure 8 shows the surface morphology of cartilage in different friction pairs. The surface damage of cartilage in ceramic/cartilage is the most serious, with obvious scratches, folds, and accumulation. The surface damage of cartilage in CoCrMo/cartilage is the second, with obvious wear marks, accompanied by ellipsoidal peeling. The surface damage of cartilage in PEEK/cartilage is the lowest, with just a relatively flat surface and only small peeling. This is because the hardness, strength, and modulus of elasticity of ceramics and CoCrMo are far greater than PEEK. In the hard–soft contact wear, the mechanical properties of materials have a more obvious impact on the tribological properties. The harder the materials are, the more obvious the damage to the soft materials is, which can be effectively avoided in the soft–soft friction pairs.

**Fig. 9:**
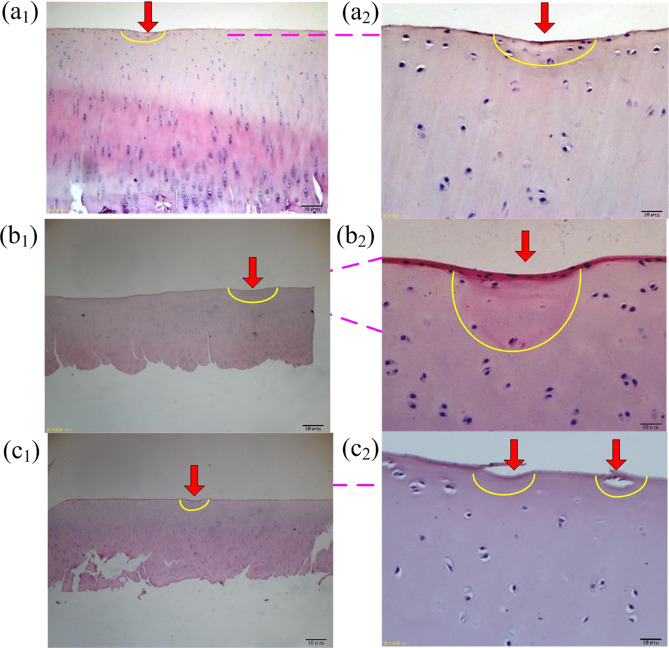
HE staining of articular cartilage surface in different friction pairs. **a1** PEEK/cartilage (×20); **a2** PEEK/cartilage (×400); **b1** CoCrMo/cartilage (×20); **b2** CoCrMo/cartilage (×400); **c1** ceramic/cartilage (×20); **c2** ceramic/cartilage (×400)

Figure 9 shows the HE staining of the worn area of cartilage surface in different friction pairs. It can be seen from the cartilage chromatogram (a1, b1, c1) at 40 magnification that the cartilage in PEEK/cartilage is less damaged than that in CoCrMo/cartilage and ceramic/cartilage. The perichondrium in CoCrMo/cartilage and ceramic/cartilage is thinner, with obscure tidal lines and hypertrophic chondrocytes, while the cartilage in PEEK/cartilage has an obvious tidal line. From the cartilage chromatogram (a2, b2, c1) at 400 magnification, for ceramic/cartilage, the perichondrium is exfoliated, the chondrocytes are hypertrophic, the nucleus of several chondrocytes is lightly stained or they have disappeared, and only a few chondrocytes are left in the worn area. For CoCrMo/cartilage, vacuolation of chondrocytes is common, and the nuclei of a few chondrocytes disappear in the worn area. For PEEK/cartilage, the surface of the cartilage is less deformed and damaged, the morphology of chondrocytes is complete, and only a few chondrocytes are hypertrophic. Considering the thickness and integrity of perichondrium and the morphology of chondrocytes in worn areas, the cartilage damage in PEEK/cartilage is the lowest, which is more conducive to the growth of chondrocytes and the development of bone.

**Fig. 10:**
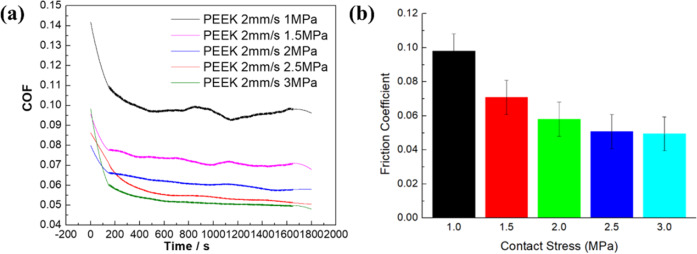
The friction coefficient of different stresses. **a** Time-varying friction coefficient; **b** stable friction coefficient

Figure 10 shows the friction coefficient of PEEK/cartilage under different stresses. Under any stress, the time-varying friction coefficient decrease sharply then stabilizes (Fig. 10a); the greater the stress, the more stable later in the period. Furthermore, the stable friction coefficient decreases with the increase of stress (Fig. 10b). When PEEK is in initial contact with cartilage, the contact surface is characterized by roughness and poor mechanical engagement, which makes the friction coefficient higher. With the sliding friction, the surface is in good contact, and with the interaction of cartilage liquid-phase-bearing and -lubricating fluid, a lubricating film is formed, which reduces the friction coefficient. Afterward, due to the self-lubricating and self-repairing function of cartilage, the friction coefficient stabilizes. Because of the unique viscoelastic properties of cartilage, the real contact area between cartilage and PEEK increases with the increase in normal stress, and the real contact area increases faster than the increase in stress, so the shear stress between contact interfaces becomes smaller. This is also the reason why the friction coefficient decreases with the increase in stress.

**Fig. 11:**
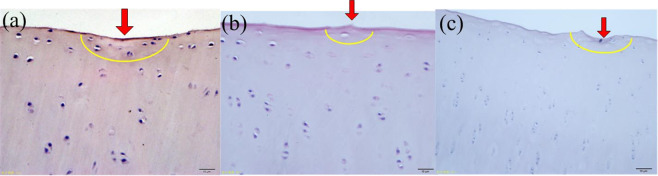
HE staining of PEEK/cartilage under different stress. **a** 1 MPa; **b** 2 MPa; **c** 3 MPa

Figure 11 shows the HE staining of PEEK/cartilage under different stresses. It can be seen that with the increase of stress, the cartilage staining decreases, the damage of cartilage surface peeling and tearing becomes more severe, the thickness of periosteum significantly decreases, the frequency of cell nucleus desalination and disappearance increases, and the cell vacuolation is more severe.

**Fig. 12:**
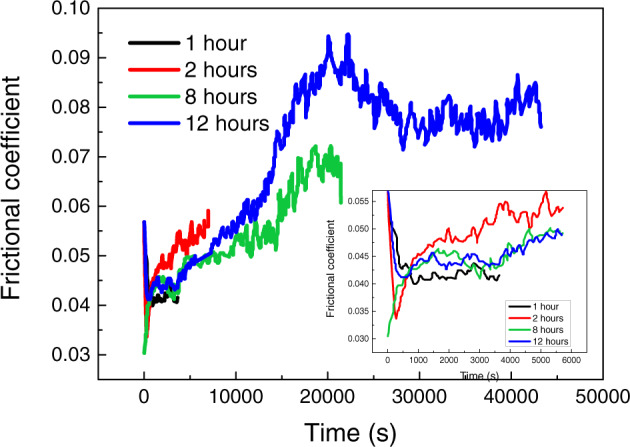
The friction coefficient of PEEK/articular cartilage with different friction times

Figure 12 shows the friction coefficient of PEEK/articular cartilage with different friction times. The friction coefficient is in a stable period from 0.3 to 1 h, increases sharply 1–5.5 h, and then decreases slowly and gradually stabilizes. In the initial stationary stage, the friction coefficient tends to be stable initially because of the uniform loading on the superficial layer of cartilage in response to the hydraulic loading mechanism of cartilage. However, with the friction, the lubrication film on the cartilage and PEEK surface is gradually destroyed, resulting in the friction coefficient increasing sharply. Then, some of the surface wear particles or exfoliated materials wash away from the surface under the lubricant washing, and some adhere to the sample surface under loads, which makes the wear surface smooth and the friction coefficient stabilizes gradually.

**Fig. 13:**
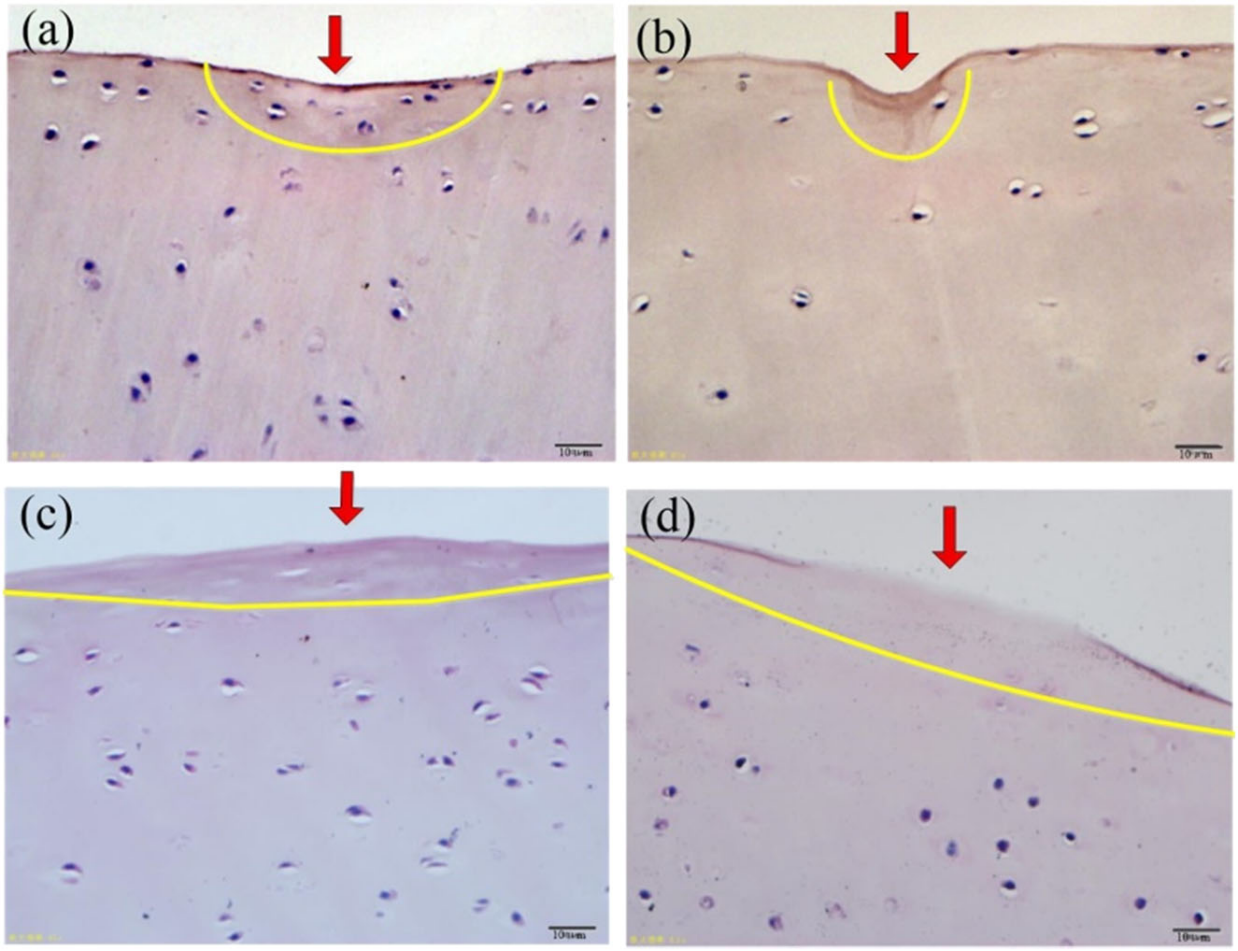
The HE staining of PEEK/cartilage with different friction times. **a** 1 h; **b** 2 h; **c** 6 h; **d** 12 h

Figure 13 shows the HE staining of PEEK/cartilage with different friction times. The longer the friction time, the larger the damaged area, and the more severe it is. For 1 h testing (Fig. 13a), the worn area of the cartilage surface is sunken. The perichondrial membrane becomes thinner due to the cartilage matrix dissolving, and some nuclei fade or disappear, but there are still a large number of cartilage cells. For 2 h (Fig. 13b), there is a deeper wear pit and a thinner perichondrium with lighter staining. The chondrocytes become hypertrophic with common vacuolization. For 6 h (Fig. 13c), the cartilage membrane was fuzzy and accompanied by peeling loss. A large number of chondrocytes experienced nuclear desalination or disappeared with general nuclear cavitation, and only a small number of chondrocytes remained in the wear area. The cartilage matrix dissolved and the staining became lighter. For 12 h (Fig. 13d), in the wear area, the perichondrium was exfoliated and lost with light staining and only a few chondrocytes. This is because for fresh cartilage, the biological activity of chondrocytes is high, and the cartilage membrane is thick. But with the friction, the cartilage membrane is gradually destroyed and shed, the cartilage layer is seriously dehydrated, and the chondrocyte activity is decreased.

### Damage mechanism

**Fig. 14:**
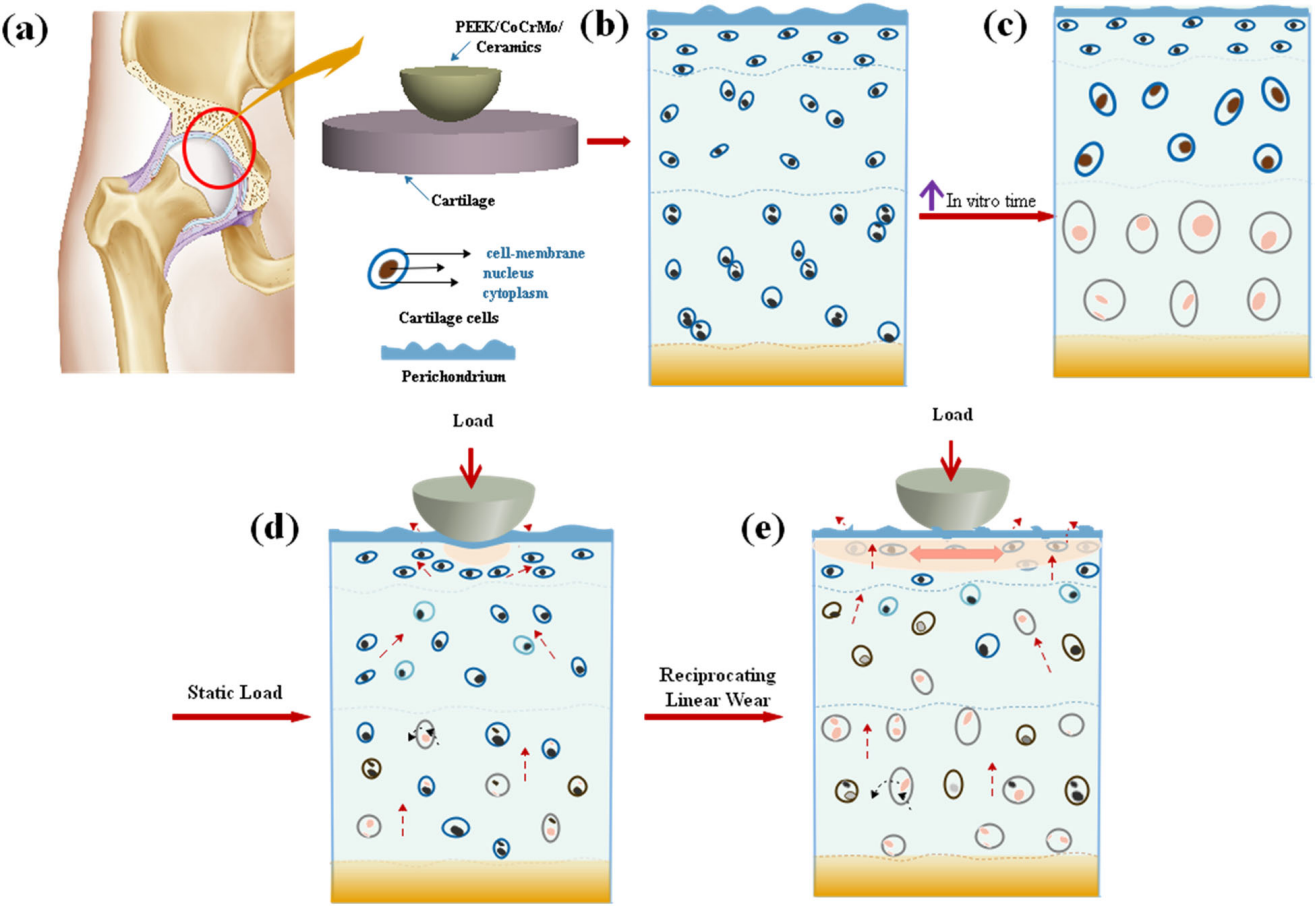
The cartilage injury mechanism

The articular surface of the natural joint is covered by a layer of articular cartilage, mainly composed of chondrocytes, extracellular matrix, and water. The cartilage structure is heterogeneous, nonlinear, anisotropic, and time-dependent. The content of chondrocytes in articular cartilage is about 1–2%, and the size, shape, and water content of chondrocytes change with the depth of cartilage [16]. So, the natural articular cartilage can be divided into four parts along its depth: superficial, middle, deep, and calcified layers, with different sizes, shape, and distribution of chondrocytes in different layers. As shown in Figs. 3a and 14b, it can be seen that the boundary between the perichondrium and the deep part of the cartilage is clear, and the perichondrium is thick (6.20 ± 0.60 μm). The chondrocytes in the superficial area are immature, with small volume and oval shape, which are parallel to the surface of cartilage in the long axis. The chondrocytes in the middle layer are mainly oblate, with a larger volume, disordered distribution direction, and some cells gathered. The chondrocytes in the deep area are mainly distributed perpendicular to the cartilage surface with large volume, and the deeper the depth, the larger and rounder the unit. Some cells have multiple nuclei and the cell aggregation is obvious.

The biological characteristics of cartilage are time-dependent, and the time of cartilage in vitro has a significant impact on the activity of chondrocytes. As shown in Fig. 14c, with the increase of the time of cartilage in vitro, the thickness of the cartilage membrane and the activity of chondrocytes decrease, and some chondrocytes become fat and large with a fuzzy nucleus. Especially in the deep area, many chondrocyte cell nuclei are desalinated or disappear. This is because the content of tissue fluid in articular cartilage accounts for about 60–85% of the total cartilage mass. With the increase of time in the incubator, the water in the cartilage layer is continuously lost and the cartilage membrane becomes thinner. After 3 h, the thickness of the cartilage membrane is only 1.52 ± 0.42 μm, which is about 70% less than that of fresh cartilage. At the same time, collagenase is effectively activated at 37 °C, the best temperature for the enzyme to generate chemical catalysis, and then the macromolecules in the cells are decomposed. They cannot get the nutrition supply of the living body, resulting in the nutritional disorder and the dissolution of the cartilage matrix. Therefore, fresh cartilage should be selected in such a case.

Natural articular cartilage is a two-phase porous viscoelastic material with high water content. Under fixed loading or deformation, the articular cartilage is mainly carried by the liquid phase at the initial stage [3] and then gradually carried by the solid matrix with the redistribution of the liquid phase. As shown in Fig. 14d, under constant loading, when the load is applied to the cartilage surface, the liquid phase exudes freely from the cartilage surface until the cartilage deformation and liquid-phase loss reach a balance, and the flow exudation stops. At this time, the stress is exerted by the balance of the solid matrix [17] and, with the increase in time, the chondrocytes change accordingly. The perichondrium becomes thinner, and the contact area has obvious deformation, with the cells in the superficial layer and the middle layer converging. Some cells became hypertrophied and the nuclei weakened. Under the loading of the solid matrix, the chondrocytes in the deep zone move vertically to the surface of the cartilage. With the increase in time, the nuclei desalt and vacuolation occurs.

Krishnan has shown that the friction coefficient of cartilage materials is related to the interaction of the liquid and solid phases in cartilage, which is specifically manifest in the change of the pressure between the spaces of the liquid in porous media and the change of the liquid-phase bearing capacity of cartilage [18]. Kai shows that the surface liquid flow, pore pressure, and stress distribution of natural cartilage have a great impact on the bearing capacity of natural cartilage materials [19]. As shown in Fig. 14e, under the effects of load and linear reciprocation, articular cartilage is mainly carried by the liquid phase between tissues in the initial stage, but with the progress of wear, the cartilage membrane is gradually damaged by shear stress and micro-convex, resulting in the acceleration of fluid loss, the weakening of liquid-phase buffer capacity, and the decrease of bearing capacity. At the same time, the cartilage deformation and the bearing capacity of the solid matrix increase. Furthermore, under the reciprocating shear stress, damage on the surface of cartilage accumulates, and the surface micro pits and micro bulges develop into tears and wear debris because the liquid-phase-bearing lubrication on the surface of the cartilage membrane cannot fully respond to the deformation of cartilage. Then the solid-phase bearing is increased, and the chondrocytes are further damaged. The activity of chondrocytes decreased rapidly, the cytoplasm dissolved, the cells became hypertrophied, and the nucleus gradually weakened and disappeared.

## Conclusions

1. With the increase of in vitro time, the perichondrium becomes thinner, denuded, and eventually lost. The cartilage matrix dissolves and the nucleus desalinates. The creep of articular cartilage increases with the increase of in vitro time, which indicates that the longer the time, the worse the anti-strain ability. Therefore, fresh cartilage should be used in experimental cartilage samples, and the time in vitro should be reduced as much as possible to maintain cartilage activity and performance.
2. The creep results of different contact pairs showed that the strain of articular cartilage in ceramic/articular cartilage was the largest, followed by CoCrMo alloy/articular cartilage, and PEEK/articular cartilage.
3. The friction coefficient for ceramic/articular cartilage is the smallest, and that of PEEK/articular cartilage is equivalent to that of CoCrMo alloy/articular cartilage. The coefficient friction PEEK/articular cartilage decreases with an increase in loads. The friction coefficient is stable at first, then sharply increases, then slowly decreases, and finally stabilizes.
4. The articular cartilage was damaged by friction testing, mainly manifested by the peeling off of the cartilage membrane and the death of chondrocytes in the friction area. The greater the contact load, the more severe the cartilage damage. Among the three friction pairs, the damage of PEEK/articular cartilage was the smallest, followed by CoCrMo alloy/articular cartilage, and ceramic/articular cartilage, indicating that the damage of soft–soft friction pairs was lower than that of hard–soft friction pairs.
